# Increased Psychological Distress, Loneliness, and Unemployment in the Spread of COVID-19 over 6 Months in Germany

**DOI:** 10.3390/medicina57010053

**Published:** 2021-01-09

**Authors:** Shuyan Liu, Stephan Heinzel, Matthias N. Haucke, Andreas Heinz

**Affiliations:** 1 Department of Psychiatry and Psychotherapy, Campus Charité Mitte, Charité—Universitätsmedizin Berlin, 10117 Berlin, Germany; hauckema@zedat.fu-berlin.de (M.N.H.); andreas.heinz@charite.de (A.H.); 2 Department of Education and Psychology, Clinical Psychology and Psychotherapy, Freie Universität Berlin, 14195 Berlin, Germany; stephan.heinzel@fu-berlin.de

**Keywords:** mental health and wellbeing, perceived social isolation, national and international survey, across cultures and over time, Sino-German assessment and evaluation, prevention policies, management in epidemics and pandemics, jobs and economic transformation, workforce recovery strategy

## Abstract

*Background*: The COVID-19 pandemic poses a challenge to global mental health. Loneliness and isolation may put people at higher risk for increased psychological distress. However, there is a lack of research investigating the development of COVID-19-related distress over time. *Materials and Methods*: We undertook an online survey among general population (N = 1903) in Germany throughout 6 months from the peak transmission period in April to the off-peak period by September 2020. *Results*: We found that the average prevalence of psychological distress caused by the COVID-19 pandemic significantly rose from 24% to 66% between the peak and off-peak transmission period, respectively. Unemployment rate and loneliness increased negative mental health outcomes, although the number of active COVID-19 cases decreased from April to September. Psychological distress scores increased mostly in female, young, and lonely people. *Conclusions*: Our results underline the importance of considering innovative alternatives to facilitate employment opportunities, distant contacts, and self-help over the course of the pandemic. Our study highlights the urgent need to pay attention to mental health services specifically targeting female, young, unemployed, and lonely people.

## 1. Introduction

The current COVID-19 pandemic has a massive impact on global mental health [1,2,3], causing sudden lifestyle changes through social distancing and isolation at home, with severe social and economic consequences [4,5]. Scientists urge mental health research to be central to the social contexts that affect the assessment and treatment of pandemics within and across countries [6,7].

A first wave of pioneering mental health surveys regarding COVID-19 were launched in January and February 2020 in China [8,9,10]. Among those studies, Shanghai Mental Health Center designed a COVID-19 Peritraumatic Distress Index (CPDI) questionnaire to assess psychological distress among the general population in China [10]. As the worldwide COVID-19 cases dramatically increased, several countries initiated their own nationwide mental health surveys between the end of March and the middle of May 2020 [10,11,12,13,14,15,16]. They used the same CPDI design, employed previously in China [10], to assess mental status of the general population during this period of peak transmission of COVID-19 [10,11,12,13,14,15,16]. The results show that the general population across countries experienced psychological distress and that the prevalence of psychological distress varied from low (11.5% (N = 410) in Nepal [11]) to mild and moderate (24.1% (N = 1007) in Germany [12] and 28.6% (N = 1035) in Italy [13]) and to high (61.1% (N = 1058) in Iran [14] and 70.8% (N = 638) in Brazil [15]) compared to 34.4% (N = 52730) in China [10]. These studies yielded consistent findings that psychological distress and challenges to well-being during the outbreak arose in cross-cultural settings [10,11,12,13,14,15,16].

In addition to the prevalence of psychological distress across cultures and countries, a systematical review by Xiong et al. (2020) showed that, from COVID-19 inception to 17 May 2020, the general population also reported relatively high rates of symptoms of anxiety (6.33% to 50.9%), depression (14.6% to 48.3%), and posttraumatic stress disorder (7% to 53.8%) [17]. Risk factors associated with distress measures were female gender, younger age group (≤40 years), presence of chronic/psychiatric illnesses, unemployment, student status, and frequent exposure to social media/news concerning COVID-19 [17]. These results suggest that there is a need to examine mental health status over the course of the pandemic in individual countries and to identify predictors of mental health among people at risk of distress from COVID-19. Specifically, unemployment increases the risk of impaired mental health, especially when the economy shrinks with very limited employment opportunities [18,19,20]. An economic downturn can trigger a fear of job loss and can widen the risk of social exclusion that may worsen the mental health [21,22,23]. Therefore, unemployment is a global public health concern in the time of COVID-19 [24].

Moreover, loneliness (or perceived social isolation) has adverse consequences for mental health, aggravating anxiety, depression, and stress-related symptoms [25]. Social distancing, isolation, quarantine, and community containment play key roles in reducing the transmission of COVID-19 [26]. However, there may be a high cost associated with these strategies that lead to elevated levels of loneliness, which in turn result in poor mental and physical health [25], including disrupted sleep, depression, anxiety, suicidal thoughts, cardiovascular disease, and immune dysfunction [27]. 

Prior to the COVID-19 pandemic, loneliness was described as a “modern behavioral epidemic” [28] with a prevalence across Europe, the U.S., and China (10–40%) [29,30]. In Germany, 10.5% of respondents reported some degree of perceived social isolation in 2017 [31]. A recent study shows that daily loneliness in Germany increased during the first two weeks of the COVID-19 lockdown starting from 16 March 2020 but decreased thereafter during the third and fourth weeks of the lockdown until 12 April 2020 [32]. Psychological distress in Germany was associated with social isolation [33], suggesting that loneliness may be increasing psychological distress. Surprisingly, Americans showed remarkable resilience in response to the initial stage of social distancing and restriction measures between February and April 2020. Researchers found only a slight increase in feelings of loneliness in the U.S., from 11% in 2018 to 13.8% in 2020 [34], without even a significant effect in average level [35].

However, that does not mean loneliness has stopped being a public health concern. There is a need to remain vigilant and to continue to monitor loneliness as the social distancing measures continue. Intermittent social distancing may remain in place until 2022 [36]. Moreover, any attempt to understand the change in loneliness must take the dimensions of time, context, and individual differences into account.

In our study, we aim to investigate the mental health status among the general population in Germany over the course of the pandemic. We undertook an online survey assessment from the peak transmission period in Germany in April 2020 for 6 months. We compared the distress level between peak (in April) and off-peak (from May to September) transmission periods of COVID-19. We identified if loneliness predicts mental health among people at risk of distress from COVID-19. The ultimate aim of our study is to identify high-risk individuals and to provide targeted care. 

Accordingly, we expect that self-reported psychological distress would increase during the pandemic due to increasing joblessness as an indicator of an economic downturn rather than the number of active COVD-19 cases per se. We also hypothesized that loneliness predicts self-reported distress, in that greater loneliness predicts more serious psychological distress. We explored the effects of age and gender that may predict psychological distress.

## 2. Materials and Methods

### 2.1. Participants and Procedure

We conducted an anonymous online survey on the Siuvo platform (https://www.siuvo.com). The Siuvo platform is an expert-level artificial intelligence platform for psychological assessment in healthcare settings. We distributed our survey using a QR code and shared it primarily via social media, advertisements, and newsletters. We collected data in Germany during the peak period of active cases of COVID-19 (i.e., by removing deaths and recoveries from total cases; ref: https://www.worldometers.info/coronavirus/country/germany/) in April 2020 and during the off-peak period from May to September 2020.

Our online survey consisted of a sociodemographic assessment (i.e., age, gender, and year of education), the COVID-19 Peritraumatic Distress Index (CPDI), and the short-form UCLA Loneliness Scale (ULS-8) questionnaires. There are several tools to measure the psychological impact of the COVID-19 pandemic [37]. We chose the CPDI scale because it has been a pioneering instrument for measuring psychological distress among general populations. It has been culturally and linguistically validated in a large range of studies across countries, including in Germany [10,11,12,13,14,15,16]. The longitudinal outcome can be better assessed if standardized scales are applied over time to better understand the impact of the pandemic. The study was approved by both the Ethics Committee of Charité – Universitätsmedizin Berlin (ref: EA2/143/20) and the Ethics Committee of Freie Universität Berlin (ref: 030/2020). 

### 2.2. Self-Reported Psychological Distress

We used the COVID-19 Peritraumatic Distress Index (CPDI) questionnaire to capture peritraumatic psychological distress during the COVID-19 pandemic [10]. The CPDI was developed by psychiatrists from the Shanghai Mental Health Center, and we used a German version [12]. The CPDI has 24-items that are rated on a 5-point scale ranging from 0 (“strongly disagree”) to 4 (“strongly agree”). A score below 28 indicates no distress, a score between 28 and 51 indicates mild to moderate distress, and a score above 52 indicates severe distress (see Supplementary Materials Table S1).

### 2.3. Measures of Loneliness

We used the German version [38] of the short-form UCLA Loneliness Scale (ULS-8) to measure an individual’s subjective perception of loneliness or social isolation [39] (see Supplementary Materials Table S2).

### 2.4. Data Analysis

Statistical tests of the CPDI and ULS-8 questionnaires were performed using SPSS Statistics Version 26 (SPSS Inc., Chicago, IL, USA). Differences were considered statistically significant at *p* < 0.05 and highly statistically significant at *p* < 0.001. We used a one-way ANOVA to assess psychological responses over time for each of the six time periods. In the ANOVA, we used “month” (April, May, June, July, August, and September) as an independent variable and the respective “CPDI score” as a dependent variable. We also tested sociodemographic differences between the six-month groups in order to control its potential confounding effect. To test whether change in loneliness can account for the change in distress across the months, we performed an analysis of covariance and used “month” as a fixed factor and “loneliness score” as a covariate and we also explored their interactions. To further investigate if such changes are associated with the sociodemographic variables (i.e., gender, age, and years of education), we conducted a multiple linear regression analysis in R version 4.0.3. (www.r-project.org). We built a first model with “ULS-8 loneliness scores”, “month”, “gender”, “age”, and “years of education” as predictors and “CPDI distress scores” as the outcome and a second model with “month”, “gender”, “age”, and “years of education” as predictors and “ULS-8 loneliness scores” as the outcome. The assumptions for both models were visually checked. An independent *t*-test was used for pairwise comparisons of the respective peak (in April) and off-peak (from May to September) periods, high versus low levels of loneliness groups, males versus females, elder versus younger groups, and high versus low levels of education groups (two-tailed *p* values were assumed).

We tested the hypotheses that (1) there is an increase in self-reported psychological distress over the course of the pandemic, (2) the increase in self-reported distress is significantly associated with joblessness as an indicator of an economic downturn, and (3) lonely persons feel more distressed. We explored whether the number of active COVID-19 cases as an indicator of the risk to become infected was correlated with distress and loneliness, and we explored the effects of age and gender.

## 3. Results

### 3.1. Group Description

From April to September 2020, 1903 respondents in Germany (1437 females; age range: 18–81, mean = 38.32, SD = 13.40) participated in our survey. Each participant completed the survey only once. The participants’ sociodemographic variables and group comparisons are presented in Table 1. With regard to sociodemographic variables between the six-month groups, we did not find a significant difference in “gender” (*F* (5, 1897) = 0.98, *p* = 0.431) but we found significant differences in “age” (*F* (5, 1889) = 43.55, *p* < 0.0001) and “education” (*F* (5, 1828) = 30.00, *p* < 0.0001) among the six groups. In general, there were gender, age, and education differences in CPDI scores. Female respondents experienced relatively higher levels of psychological distress compared to males, *t* (1901) = 3.01, *p* = 0.003. Younger respondents experienced relatively higher levels of psychological distress compared to elders, *t* (1836.830) = −8.59, *p* < 0.001. Respondents with fewer years of education experienced relatively higher levels of psychological distress compared to those with more years of education, *t* (1662.836) = −9.66, *p* < 0.001, as shown in Table 1.

Over the last six months (183 days), in Germany, there have been an average of 19,630 active cases per day, which reached its peak with an average of 54,715 active cases in April (with a peak number on 6 April 2020 at 72,865 cases on this day) and an off-peak average of 6739 cases in July (down to 5882 total cases in a single day on 19 July 2020).

### 3.2. Increased Psychological Distress over the Course of the Pandemic in Germany

**Figure 1:**
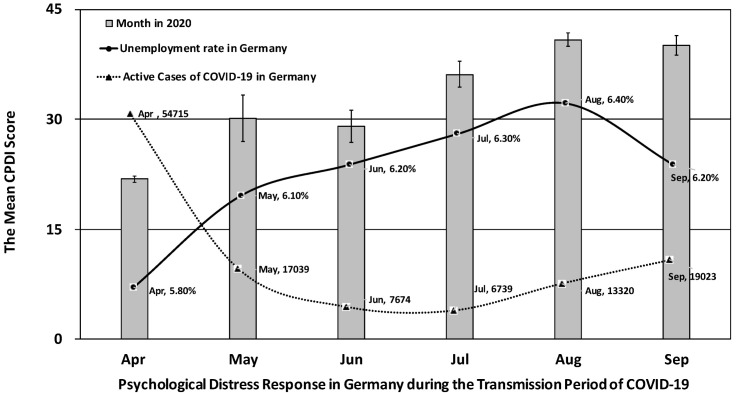
Psychological distress response, unemployment rate, and active cases of COVID-19 in Germany between April and September 2020: the error bars represent standard errors of the mean. Data of the unemployment rate in Germany were retrieved from the Federal Statistical Office of Germany (https://www.destatis.de/EN/Themes/Economy/Short-Term-Indicators/Labour-Market/arb210a.html). Date of the active cases of COVID-19 in Germany (i.e., by removing deaths and recoveries from total cases) were retrieved from Worldometer’s COVID-19 data (https://www.worldometers.info/coronavirus/country/germany/). The monthly average of active cases was calculated by the sum of daily active cases divided by the number of days in that month.

Self-reported psychological distress increased over the course of the pandemic in Germany, *F* (5, 1897) = 112.17, *p* < 0.001. There was a significant difference in psychological outcomes between the peak versus off-peak transmission period of COVID-19 in Germany, *t* (1485.474) = −22.09, *p* < 0.001. The average COVID-19 Peritraumatic Distress Index (CPDI) score for the off-peak transmission period from May to September (mean = 38.94, SE = 19.84) was 17.08 higher than the average CPDI score for the peak transmission period in April (mean = 21.85, SE = 12.64), shown in Figure 1. 

During the period of off-peak transmission of COVID-19 pandemic, 66% of respondents in Germany experienced psychological distress varying from 40% mild to 26% severe. On the other hand, during the period of peak transmission of COVID-19, only 24% of populations in Germany reported distress with 21% mild and 4% severe stress.

Interestingly, the daily average of active cases per month did not statistically significantly correlate with perceived distress (Pearson *r* = −0.658, *p* = 0.155, two-tailed). Instead, increased perceived distress over time was significantly associated with increased unemployment rate in Germany (Pearson *r* = 0.874, *p* = 0.023, two-tailed). An increase in the unemployment rate was statistically significantly associated with the prevalence of active COVID-19 cases (Pearson *r* = −0.886, *p* = 0.019, two-tailed), indicating that people could be left without work due to COVID-19, which in turn affected perceived stress levels.

### 3.3. Loneliness Predicts Psychological Distress 

We found statistically significant differences in psychological distress scores between the groups by month when adjusted for ULS-8 loneliness score, *F* (4, 890) = 11.36, partial *η*^2^ = 0.049, *p* < 0.0001, and there was no significant interaction between independent variable “month” and the covariate “ULS-8 loneliness score”, *F* (4, 886) = 1.65, partial *η*^2^ = 0.007, *p* = 0.161, indicating that perceived loneliness predicted change in psychological distress over time. Such changes were not associated with gender and age but with years of education. To test for the influence of demographics, we conducted a multiple linear regression model. The predictors “ULS-8 loneliness scores” (*b* = 2.14, *t* (853) = 25.00, *p* < 0.0001) and “month” (*b* = 2.42, *t* (853) = 4.64, *p* < 0.0001) statistically significantly increased psychological distress, whereas “years of education” (*b* = −0.67, *t* (853) = −4.99, *p* < 0.0001) significantly decreased psychological distress amid COVID-19. The factors “gender” (*b* = 1.47, *t* (853) = 1.24, *p* = 0.217) and “age” (*b* = −0.02, *t* (853) = −0.47, *p* = 0.64) did not show a statistically significant influence on psychological distress. Moreover, except for the factor “years of education” (*b* = −0.12, *t* (854) = −2.27, *p* = 0.024), the other factors “month” (*b* = 0.07, *t* (854) = 0.35, *p* = 0.723), “gender” (*b* = 0.46, *t* (854) = 0.96, *p* = 0.336), and “age” (*b* = −0.01, *t* (854) = −0.32, *p* = 0.750) did not show a statistically significant influence on “ULS-8 loneliness scores”. In general, respondents who reported more feelings of loneliness had a greater propensity to report psychological distress (Pearson r = 0.649, *p* < 0.001, two-tailed). During the period of off-peak transmission of COVID-19 pandemic, 66% of respondents in Germany reported felt lonely, varying from 42% “sometimes” to 24% “always” feeling lonely. The average ULS-8 loneliness score was 19.05, with a standard deviation of 5.89. Accordingly, the ULS-8 loneliness scores for each month were as follows: May, mean = 19.63, SD = 5.33; June, mean = 19.13, SD = 4.77; July, mean = 18.18, SD = 5.86; August, mean = 19.18, SD = 5.97; and September, mean = 19.15, SD = 6.01. 

**Figure 2:**
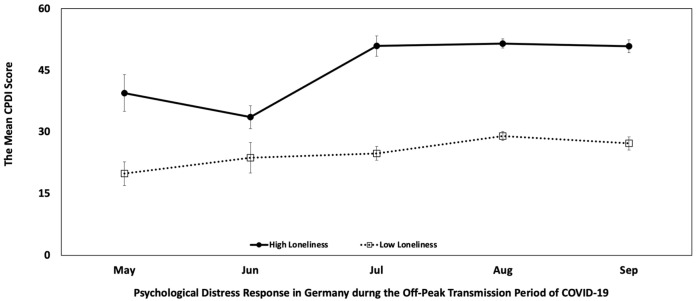
The effect of high (N = 446) versus low (N = 407) loneliness scores on psychological distress response in Germany during the period of off-peak transmission of COVID-19: for group comparison, each participant’s self-reported loneliness score (ULS-8) was ranked higher versus lower than the median (N = 853/896: 43 subjects’ data in the median were removed).

There was a significant difference in self-reported psychological distress between individuals who reported high versus low levels of loneliness, *t* (850.392) = 20.03, *p* < 0.001. The average CPDI score for individuals who reported higher levels of loneliness (mean = 49.86, SE = 17.52) was 22.68 higher than the average CPDI score for those who reported lower levels of loneliness (mean = 27.18, SE = 15.56), shown in Figure 2.

Due to a high proportion of female participants, we also conducted additional analysis of males and females separately. We also found that the same change in perceived loneliness over time predicted change in psychological distress in males and females. In male participants, the predictors “ULS-8 loneliness scores” (*b* = 2.22, *t* (197) = 12.23, *p* < 0.0001) and “month” (*b* = 3.20, *t* (197) = 2.60, *p* < 0.01) statistically significantly increased psychological distress, and in female participants, the predictors “ULS-8 loneliness scores” (*b* = 2.16, *t* (656) = 21.90, *p* < 0.0001) and “month” (*b* = 2.72, *t* (656) = 4.79, *p* < 0.0001) statistically significantly increased psychological distress. 

## 4. Discussion

### 4.1. General Discussion

This study is among the first national-sample studies to track temporal changes in population mental health from the lockdown period to the subsequent period of rather low active COVID-19 cases per months. Consistent with our hypothesis, we found an overall increase in psychological distress over 6 months’ time within a general population in Germany. Additionally, we found that this increase in psychological distress was associated with the persistent and substantial rise of unemployment rates in Germany. Moreover, our data suggests that years of education is a protective factor for psychological distress. This might be due to higher income, higher job security, or home office opportunities associated with increased level of education. Finally, we found that loneliness predicted COVID-19-related distress. 

According to the Federal Statistical Office of Germany (Destatis), the unemployment rate in Germany remained stable at 5.1% until March 2020 when restrictions were in place due to COVID-19. Since then, the unemployment rate has risen rapidly from 5.8% in April to 6.4% in August by a 0.1% monthly rise (https://www.destatis.de/EN/Themes/Economy/Short-Term-Indicators/Labour-Market/arb210a.html). Cross-sectional studies showed that unemployment was associated with increased mental health problems, depression and anxiety disorders especially among young people [18]. Unemployment insurance (i.e., government payments to eligible individuals to replace part of pre-job loss income during their job search, “Arbeitslosengeld” in Germany) may act as a “safety net” not only to protect individuals from hardship but also to enhance mental health resilience [19]. Wanberg et al. (2020) found that higher perceived unemployment insurance generosity was associated with better mental health via reduced time pressure and financial strain [19]. 

Beyond creating employment opportunities and enhancing unemployment insurance, other strategies that enable individuals to remain active in their job search included productive use of time on meaningful activities, conserving financial resources, social supports, and cognitive techniques (e.g., holding a positive outlook, reevaluating expectations, reappraisal, determination, and agency) [40]. In the era of COVID-19, researchers suggest that it is high time to design evidence-based interventions for unemployed individuals and to examine work–family balance and unemployment issues among youth [24]. 

Our data suggest that loneliness is a predictor for COVID-19-related distress. In Germany, a sharp spike in people seeking for mental health help appears to be mainly driven by anxiety, depression, and loneliness [41], and the federal states of Germany, where stricter measures were in place, such as “stay-at-home” orders in Bavaria, Saarland, and Sachsen-Anhalt, had an even stronger increase in helpline contacts [41]. This may be particularly challenging for females and young people [17,42,43]. To deal with the immediate and long-term consequences of COVID-19, it is essential to tap in the power of social community participation [44,45]. Qi et al. (2020) found that lower levels of social support were associated with a higher prevalence of mental health problems among adolescents during the COVID-19 outbreak [44]. Thus, it appears to be necessary to provide incentives and to support vulnerable individuals in response to COVID-19 [45]. For example, online communities are a way to promote social participation and tangible support by sharing online workshops, resources, and guides [24]. Internet- and mobile-based psychological testing and assessment can also capture a mental health profile of the community and can provide evidence to innovate and strengthen practice and policy amid a global pandemic [46,47]. For example, a study on the Germany’s largest free mental health helpline “TelefonSeelsorge” (https://www.telefonseelsorge.de/) showed that the demand for mental health services has been increased by approximately 20% from 1800 to 2400 contacts per day in the first week of the lockdown [41].

### 4.2. Limitations

Our current results must remain tentative due to the lack of daily and momentary state variations in psychological distress to detect more subtle effects of COVID-19-related restrictions and economic downturn on mental health. Our survey neither elicited an individual’s employment nor disentangled the causal interplay between unemployment and poor mental health. An increase in fear of unemployment may also worsen mental health [21]. Our study is also limited by a lack of validated and comparable, pre-COVID-19 baseline data against which to measure change either within individuals or across the population as a whole. In addition, pre-pandemic life circumstances may also remain important determinants of people’s mental health during the pandemic. The generalizability of results is limited to populations that share characteristics with our sample (e.g., the population has access to the Internet to complete our survey). At the time of our study, the COVID-19 crisis is still unfolding, which further limits the generalizability of our findings. We hope that the reported descriptive associations encourage replications of the research design in different settings and further theoretical elaboration.

### 4.3. Outlook and Future Perspectives

Intermittent social distancing may remain in place until 2022, and a resurgence of COVID-19 is possible until 2024 [36]. An inclusive response to and recovery from COVID-19 requires an integrated longitudinal approach to anticipate the impact across different target groups. The use of mobile digital health interventions, such as mobile health (mHealth) tools, is one possible solution to deliver the objective to mitigate negative psychosocial consequences of the COVID-19 pandemic [33]. Virtual reality exercise has also been used in mental health and wellness promotion [48]. Education, self-care, and family support should form part of mental health prevention strategies, which can involve multiagency collaboration among housing, education, and employment services, with support from the voluntary and mental health sectors [49]. These interventions could include digital forms of study groups, peer group sessions, mentoring, and psychological counseling [50]. Friendship, interaction, social support, and studying with others have been argued to impact their well-being and academic success, but they often require meeting opportunities and informal settings to develop [51]. In a long-term perspective, it is important to examine whether the rules, knowledge, attitudes, and coping styles developed during the pandemic are maintained post-pandemic. It might be a long and unclear road until the COVID-19 pandemic ends. Future studies are needed to investigate stress changes during the course of the pandemic, particularly among vulnerable groups including females and young people [17,42,43], people with chronic diseases [52], and medical professionals [53]. Integration of long-term and short-term research in response to COVID-19, recovery strategies, and resilience may help us understand what we need to do to better manage future epidemics and pandemics. 

## Figures and Tables

**Table 1:** Participants’ sociodemographic variables and group comparisons.

	Group	N	Mean Age (SEM)	*p*	Mean Education (SEM)	*p*	Mean CPDI (SEM)	*p*
Gender	Male	466	39.20 (0.65)	0.103	16.80 (0.20)	0.760	27.66 (0.86)	0.003
	Female	1437	38.03 (0.35)		16.87 (0.11)		30.63 (0.49)	
Age, years	Elder ^1^	927	49.82 (0.30)	<0.001	17.29 (0.15)	<0.001	26.38 (0.57)	<0.001
	Younger ^1^	925	26.90 (0.15)		16.35 (0.12)		33.65 (0.63)	
Education, years	Higher ^2^	801	40.15 (0.41)	<0.001	20.61 (0.10)	<0.001	25.35 (0.64)	<0.001
	Lower ^2^	903	37.03 (0.49)		13.49 (0.06)		33.64 (0.69)	

Abbreviations: *p*—statistical significance, N—number of participants, SEM—standard error of the mean, CPDI—COVID-19 Peritraumatic Distress Index (CPDI). ^1^ For the age group comparison (elder vs. younger), each participant’s age was ranked higher versus lower than the median (N = 927/925: 43 subjects’ data in the median (value of 36) were removed). ^2^ For group comparison in education levels (higher vs. lower), each participant’s years of education was ranked higher versus lower than the median (N = 801/903: 130 subjects’ data in the median (value of 17) were removed). To test for differences between the groups, independent samples *t*-test was used.

## Data Availability

The data presented in this study are available on request from the authors.

## Supplementary Materials

The following are available online at https://www.mdpi.com/1010-660X/57/1/53/s1, Table S1: The German version of COVID-19 Peritraumatic Distress Index (CPDI) questionnaire, Table S2: The German version of the short-form UCLA Loneliness Scale (ULS-8).
