# Supplementary material for: Increased Psychological Distress, Loneliness, and Unemployment in the Spread of COVID-19 over 6 Months in Germany
Source: Medicina (Kaunas). 2021 Jan 9;57(1):53. doi: 10.3390/medicina57010053 (PMC7827929; doi:10.3390/medicina57010053)
Supplement: Supplementary file 1 [file medicina-57-00053-s001.pdf]

Supplementary

Table S1. The German version of COVID-19 Peritraumatic Distress Index (CPDI) questionnaire

**Bitte geben Sie an, ob Sie den Aussagen zu den folgenden Empfindungen, Aktivitäten und Gedanken in den letzten 7 Tagen zustimmen.**	
1	Im Vergleich zu der Zeit vor der Corona-Phase fühle ich mich nervöser und ängstlicher.
2	Ich fühle mich derzeit unsicherer und/der habe viele Masken, Medikamente, Desinfektionsmittel, Handschuhe und/oder andere Haushaltsgegenstände gekauft.
3	Ich kann nicht aufhören, mir vorzustellen, dass meine Familie oder ich selbst infiziert sind, und mich deshalb ängstlich und besorgt zu fühlen.
4	Im Vergleich zu der Zeit vor der Corona-Phase fühle ich mich leerer und hilflos, egal was ich tue.
5	Ich fühle mit den COVID-19-Patienten und ihren Familien. Ich bin deshalb traurig.
6	Im Vergleich zu der Zeit vor der Corona-Phase fühle ich mich hilfloser und bin wütend über Menschen um mich herum, Behörden und Medien.
7	Im Vergleich zu der Zeit vor der Corona-Phase verliere ich vermehrt das Vertrauen in die Menschen um mich herum.
8	Ich sammle den ganzen Tag Informationen über COVID-19. Auch wenn es nicht notwendig ist, kann ich damit nicht aufhören.
9	Ich vertraue den Informationen aus unterschiedlichsten Quellen über COVID-19 rückhaltlos.
10	Ich glaube eher negativen Nachrichten über COVID-19 und bin skeptisch gegenüber guten Nachrichten.
11	Ich verbreite ständig Neuigkeiten über COVID-19 (meistens negative Nachrichten).
12	Ich vermeide aus zu großer Angst Nachrichten über COVID-19 zu sehen.
13	Ich bin derzeit gereizter und habe häufiger Konflikte mit meiner Familie.
14	Im Vergleich zu der Zeit vor der Corona-Phase fühle ich mich müder und manchmal sogar erschöpft.
15	Aufgrund von Angstgefühlen werden meine Reaktionen träge.
16	Im Vergleich zu der Zeit vor der Corona-Phase fällt es mir schwerer, mich zu konzentrieren.
17	Im Vergleich zu der Zeit vor der Corona-Phase fällt es mir schwerer, Entscheidungen zu treffen.
18	Während dieser COVID-19-Phase ist mir oft schwindelig oder ich habe Rückenschmerzen und Brustschmerzen.
19	Während dieser COVID-19-Phase habe ich oft Magenschmerzen, Blähungen und andere Magenbeschwerden.
20	Im Vergleich zu der Zeit vor der Corona-Phase fühle ich mich unwohler, wenn ich mit anderen kommuniziere.
21	Im Vergleich zu der Zeit vor der Corona-Phase spreche ich weniger oft mit meiner Familie.
22	Während dieser COVID-19-Phase kann ich nicht gut schlafen und / oder träume immer davon, dass ich oder meine Familie von COVID-19 infiziert werden.
23	Im Vergleich zu der Zeit vor der Corona-Phase habe ich weniger Appetit.
24	Im Vergleich zu der Zeit vor der Corona-Phase habe ich öfter Verstopfung oder muss häufig Wasser lassen.

Note: CPDI ≥ 52: Severe distress; CPDI between 28-51: Mild to moderate distress; CPDI < 28: No to low distress; The response scale ranges from 0 (stimme überhaupt nicht zu), 1 (stimme kaum zu), 2 (stimme weder zu noch nicht zu), 3 (stimme etwas zu), to 4 (stimme völlig zu).

Table S2. The German version of the short-form UCLA Loneliness Scale (ULS-8)

**Bitte geben Sie an, ob Sie den Aussagen zu den folgenden Empfindungen, Aktivitäten und Gedanken in den letzten 7 Tagen zustimmen.**	
1	Im Vergleich zu der Zeit vor der Corona-Phase habe ich weniger Gesellschaft.
2	Ich habe derzeit niemanden, an die ich mich wenden kann.
3	Im Vergleich zu der Zeit vor der Corona-Phase bin ich nicht mehr eine so kontaktfreudige Person.
4	Im Vergleich zu der Zeit vor der Corona-Phase fühle ich mich stärker ausgeschlossen.
5	Im Vergleich zu der Zeit vor der Corona-Phase fühle ich mich stärker von den anderen isoliert.
6	Im Vergleich zu der Zeit vor der Corona-Phase kann ich seltener mit anderen zusammen sein, wenn ich das will.
7	Im Vergleich zu der Zeit vor der Corona-Phase bin ich unglücklicher, weil ich so zurückgezogen lebe.
8	Im Vergleich zu der Zeit vor der Corona-Phase haben die anderen Menschen es schwerer, an mich heranzukommen.

Note: ULS-8 ≥ 24: Always feel lonely; ULS-8 within 16-23: Sometimes feel lonely; ULS-8 < 16: Never to rarely feel lonely; The response scale ranges from 1 (trifft nicht zu), 2 (trifft kaum zu), 3 (trifft eher zu), to 4 (trifft voll zu).
